# Supplementary material for: Cultivation-independent genomes greatly expand taxonomic-profiling capabilities of mOTUs across various environments
Source: Microbiome. 2022 Dec 5;10:212. doi: 10.1186/s40168-022-01410-z (PMC9721005; doi:10.1186/s40168-022-01410-z)
Supplement: Supplementary file 2 — Additional file 1: Supplementary Figure 1. Environment-specific membership of genomes in ref-, meta- and ext-mOTUs. A total of 499,512 genomes derived from 23 environments (environments with few genomes are grouped as ‘Other’, see Supplementary Tables 1 and 3) were used for the extension. The number of genomes was normalized by environments. The proportions of genomes per environment that are either associated with ref- and meta-mOTUs or were used to build ex-mOTUs are shown in the colors blue, green or orange, respectively. For example, the majority of genomes from the human gut match ref-mOTUs, whereas the vast majority of genomes from the fish environment are used to build ext-mOTUs. Supplementary Figure 2. Comparison of Shannon index from profiling using mOTUs and 16S rRNA gene OTUs. In order to improve our understanding, we compared the Shannon index evaluated with mOTUs (y-axis) and 16S rRNA OTUs (x-axis) reconstructed from the cattle and soil samples. Pearson correlation of indices generated from cattle profiles show a high agreement between mOTUs and 16S rRNA based methods whereas mOTUs underestimates species diversity for soil samples. The mOTUs profiles were generated using default parameters. For the 16S RNA profiles we extracted the first 100 bp from reads containing the V4 primer sequence and clustered at 97% identity using vsearch (--derep_fulllength, --cluster_size --id 0.97, --usearch_global --id 0.97). Supplementary Figure 3. OPAL score broken down to individual metrics for the 63 mouse gut metagenomic samples. The evaluation was performed using the OPAL tool [1] on 63 simulated mouse gut metagenomes [2], which also provided taxonomic profiles for seven different taxonomic profiling tools, and to which we have added mOTUs3 profiling results. The OPAL tool ranks the tools for each sample and for each taxonomic level. The measures considered are completeness, purity, L1 norm error and weighted UniFrac error, shown individually in the bottom 4 plots. T [file 40168_2022_1410_MOESM1_ESM.zip › Supplementary Information_embedded_figs.pdf]

Supplementary Information

Supplementary Information for this manuscript includes:

- Legends for Supplementary Figures 1-4
- Legends for Supplementary Tables 1-6

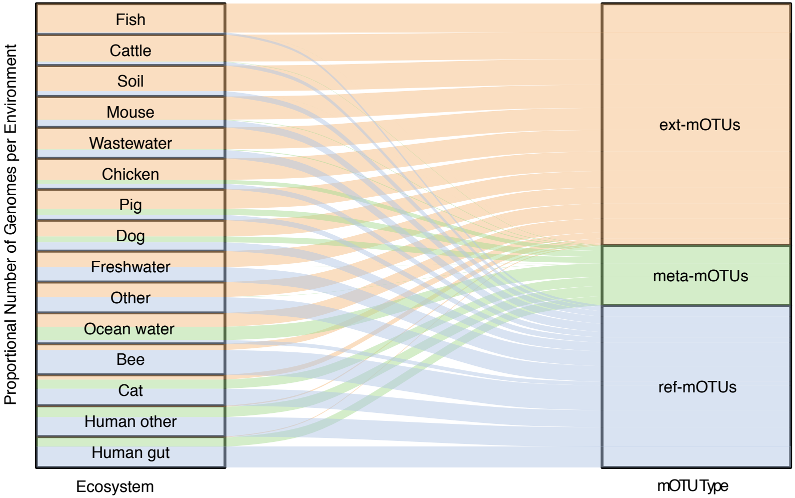
Proportional Number of Genomes per Environment
Fish
Cattle
Soil
Mouse
Wastewater
Chicken
Pig
Dog
Freshwater
Other
Ocean water
Bee
Cat
Human other
Human gut
ext-mOTUs
meta-mOTUs
ref-mOTUs
Ecosystem
mOTU Type

Supplementary Figure 1. Environment-specific membership of genomes in ref-, meta- and ext-mOTUs. A total of 499,512 genomes derived from 23 environments (environments with few genomes are grouped as ‘Other’, see Supplementary Tables 1 and 3) were used for the extension. The number of genomes was normalized by environments. The proportions of genomes per environment that are either associated with ref- and meta-mOTUs or were used to build ex-mOTUs are shown in the colors blue, green or orange, respectively. For example, the majority of genomes from the human gut match ref-mOTUs, whereas the vast majority of genomes from the fish environment are used to build ext-mOTUs.

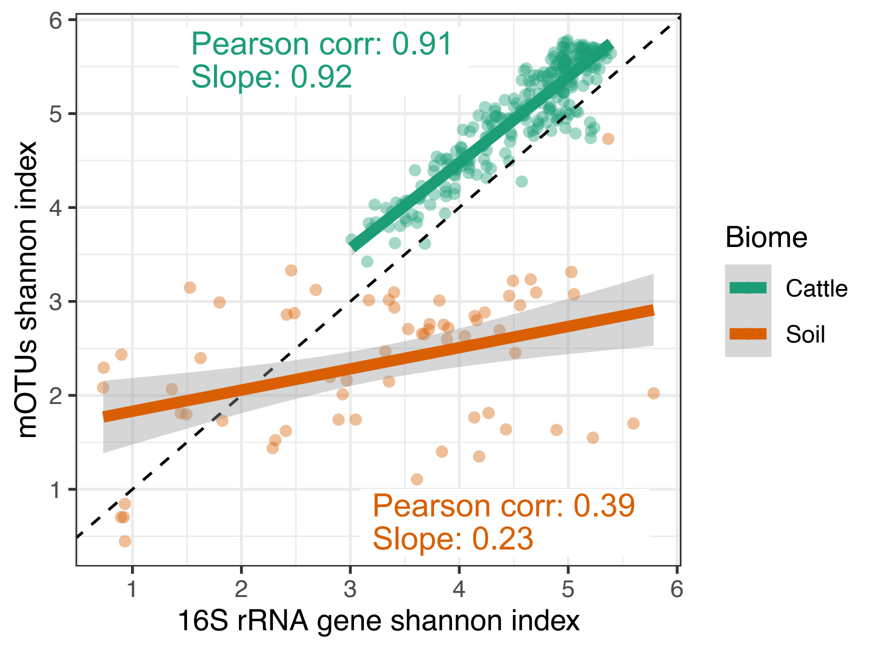
Pearson corr: 0.91
Slope: 0.92
Pearson corr: 0.39
Slope: 0.23
mOTUs shannon index
16S rRNA gene shannon index
1
2
3
4
5
6
Biome
Cattle
Soil

Supplementary Figure 2: Comparison of Shannon index from profiling using mOTUs and 16S rRNA gene OTUs. In order to improve our understanding, we compared the Shannon index evaluated with mOTUs (y-axis) and 16S rRNA OTUs (x-axis) reconstructed from the cattle and soil samples. Pearson correlation of indices generated from cattle profiles show a high agreement between mOTUs and 16S rRNA based methods whereas mOTUs underestimates species diversity for soil samples. The mOTUs profiles were generated using default parameters. For the 16S RNA profiles we extracted the first 100 bp from reads containing the V4 primer sequence and clustered at 97% identity using vsearch (*--derep_fulllength, --cluster_size --id 0.97, --usearch_global --id 0.97*).

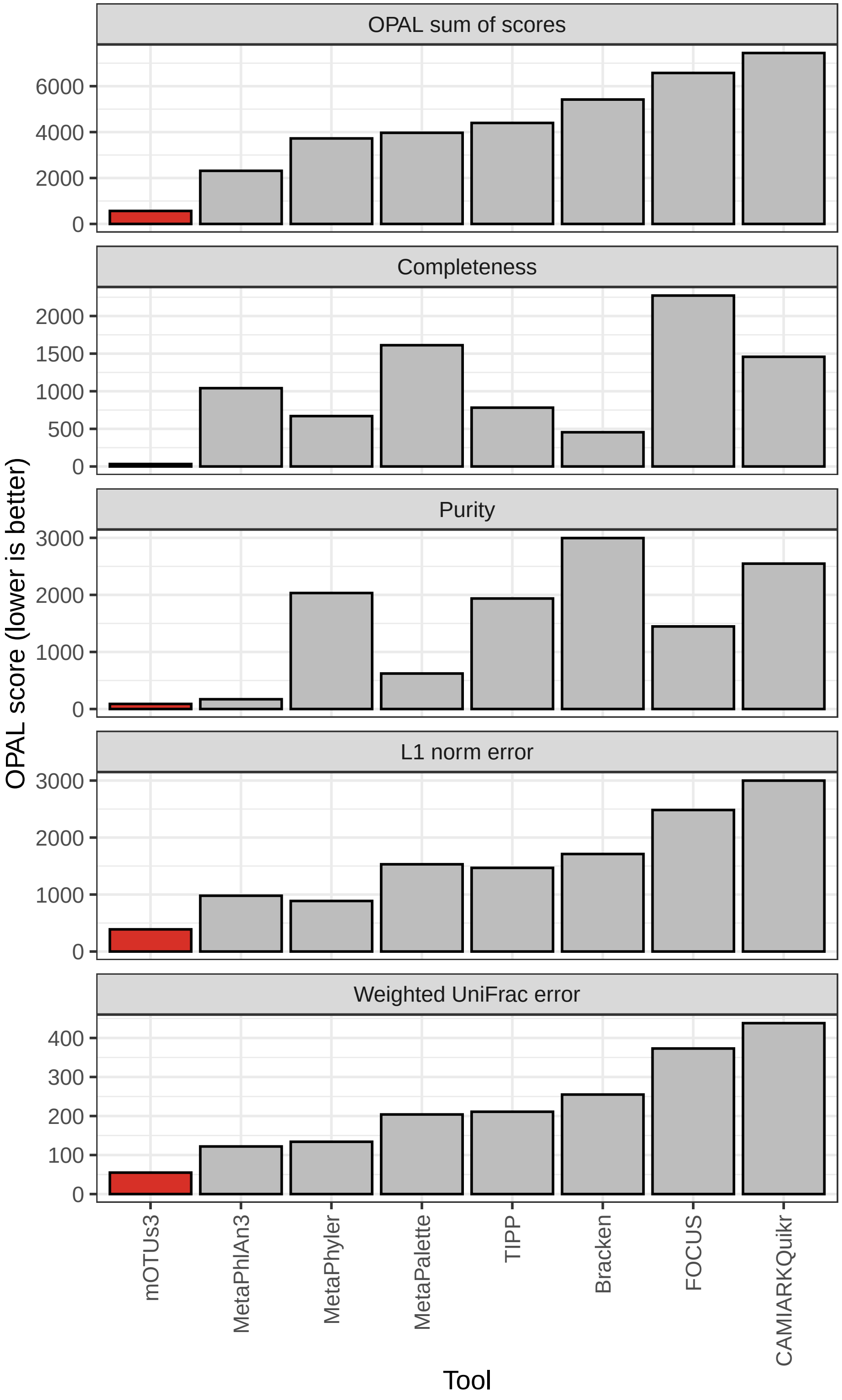

OPAL sum of scores
Completeness
Purity
L1 norm error
Weighted UniFrac error
OPAL score (lower is better)
Tool
mOTUs3
MetaPhlAn3
MetaPhyler
MetaPalette
TIPP
Bracken
FOCUS
CAMIARKQuikr

Supplementary Figure 3. OPAL score broken down to individual metrics for the 63 mouse gut metagenomic samples. The evaluation was performed using the OPAL tool [1] on 63 simulated mouse gut metagenomes [2], which also provided taxonomic profiles for seven different taxonomic profiling tools, and to which we have added mOTUs3 profiling results. The OPAL tool ranks the tools for each sample and for each taxonomic level. The measures considered are completeness, purity, L1 norm error and weighted UniFrac error, shown individually in the bottom 4 plots. Tools with a lower score perform better, as the OPAL score is a sum over rank. The top plot represents the OPAL sum of scores, which is the sum over the four individual measures. mOTUs3 scored best in all categories, including the OPAL sum of scores.

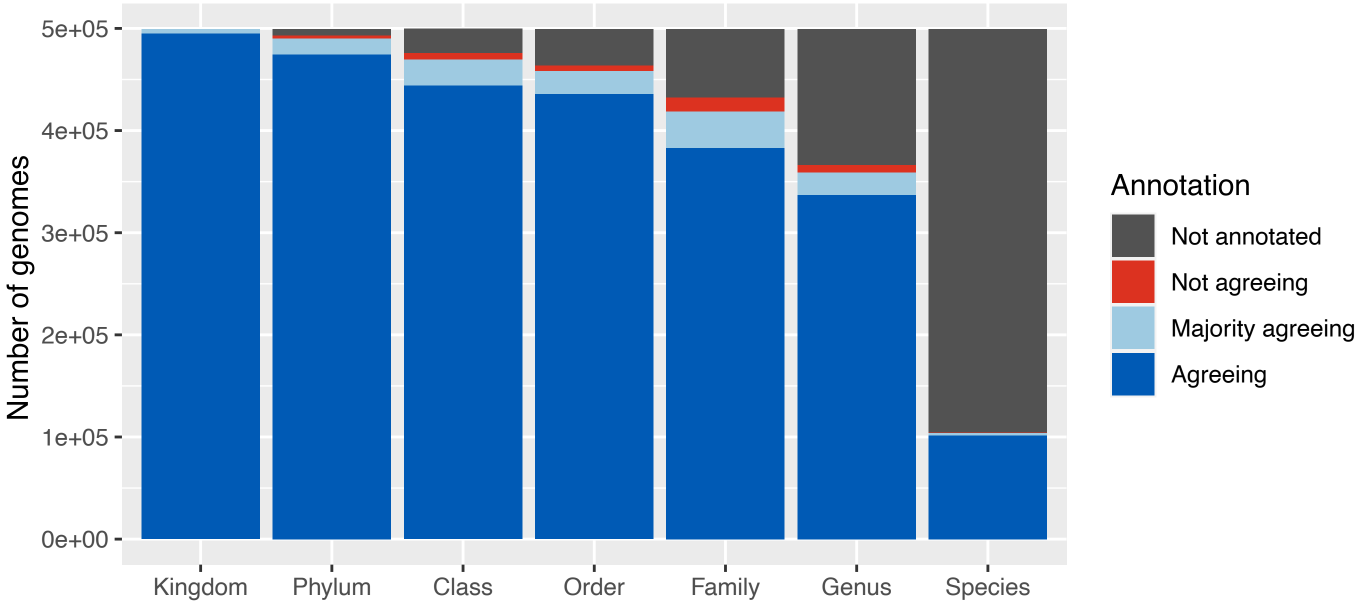

Percentage of not agreeing

	Kingdom	Phylum	Order	Class	Family	Genus	Species
**To total (499,512 genomes)**	0	0.61	1.32	1.1	2.76	1.43	0.07
**To annotated genomes for this tax. level**	0	0.62	1.38	1.19	3.18	1.96	0.32

Supplementary Figure 4. Taxonomic consistency of marker genes from the 499,512 genomes used to extend the mOTUs database. Marker genes from each genome were taxonomically annotated to evaluate taxonomic consistency. Agreeing, all marker genes have the same annotation; Majority agreeing, more than half of the marker genes agree to one taxonomic annotation; Not agreeing, there is no taxonomic annotation that agrees in more than 50% of the marker genes; Not annotated, there is no taxonomic annotation for this taxonomic level. Below the graph, we show a table with the percentage of Not agreeing annotations per taxonomic level, either as the percentage of all genomes (top) or of the genomes that have an annotation at that taxonomic level (bottom).

Supplementary Table Legends

Supplementary Table 1: Included studies and associated environments.

Data from 91 studies from 23 environments were included in the extension and/or profiling of the mOTUs database. Of these, 39 studies were selected for in-house MAG reconstruction and 11,164 sequencing samples from 67 studies were used for taxonomic profiling.

Supplementary Table 2: Sequencing samples included in the taxonomic profile.

A total of 11,164 samples were taxonomically profiled. Sample names are connected to public repositories by biosample and sequencing run ids. The project name column links the sample name to the study name used in Supplementary Table 1.

Supplementary Table 3: Breakdown of taxonomic novelty in ext-mOTUs.

Taxonomic novelty increases with higher ranks, i.e., more than 50% of ext-mOTUs were assigned to previously unknown families.

Supplementary Table 4: Contribution of genomes to ref-, meta- or ext-mOTUs.

Genomes/MAGs from different studies and environments contribute in varying proportions to the extension of the database.

Supplementary Table 5: Data for Figure 1.

For each sample that passed the filter (total 5,756), we reported the relative abundance for each mOTU type. Additionally, we added the total number of detected mOTUs and the habitat.

Supplementary Table 6: Data for Figure 5a.

Generalized fold change and adjusted p-value for species detected in 20 sheep rumen metagenomes when profiled with mOTUs3, Bracken or MetaPhlAn3.

References

1. Meyer F, Bremges A, Belmann P, Janssen S, McHardy AC, Koslicki D. Assessing taxonomic metagenome profilers with OPAL. Genome Biol. 2019;20:51.

2. Meyer F, Lesker T-R, Koslicki D, Fritz A, Gurevich A, Darling AE, et al. Tutorial: assessing metagenomics software with the CAMI benchmarking toolkit. Nat Protoc. 2021;16:1785–801.
